# Single-Session Treatment of Upper Extremity Deep Venous Thrombosis and Central Venous Catheter Malfunction Using the ClotTriever System

**DOI:** 10.7759/cureus.12071

**Published:** 2020-12-14

**Authors:** Siddharth Agarwal, Christopher Sosnofsky, Jamie Saum, Manu Aggarwal, Sandeep Patel

**Affiliations:** 1 Medicine, Vardhman Mahavir Medical College and Safdarjung Hospital, New Delhi, IND; 2 Cardiology, Heritage College of Osteopathic Medicine, Athens, USA; 3 Interventional Cardiology, Mercy Health St. Rita's Medical Center, Lima, USA; 4 Interventional Cardiology, Vein Care Center, Lima, USA

**Keywords:** percutaneous mechanical thrombectomy, breast cancer, catheter directed thrombolysis, anti-coagulation

## Abstract

Intravenous catheters account for the majority of cases of upper extremity deep vein thrombosis (UEDVT), with a higher incidence in patients suffering from malignancy. Sequelae of UEDVT are similar to that of lower extremity DVT, comprising post-thrombotic syndrome and pulmonary embolism. While there are several treatment options for UEDVT including systemic anticoagulation, catheter-directed thrombolysis, and percutaneous mechanical thrombectomy, due to the absence of consistent guidelines regarding its management, treatment is often individualized based on patient characteristics, clinical factors, and technical considerations. We present a case of a 49-year-old female suffering from breast cancer with a central venous catheter (CVC) who came to the clinic with UEDVT and CVC malfunction and was successfully treated with mechanical thrombectomy using the ClotTriever System (Inari Medical, Irvine, CA). To our knowledge, this is the first report of the ClotTriever System being used to treat UEDVT and simultaneously salvage the CVC in a single session.

## Introduction

The prevalence of upper extremity deep vein thrombosis (UEDVT) has been on the rise for the past decade but still remains an underdiagnosed entity due to a significant percentage of asymptomatic patients. The etiology of UEDVT is overwhelmingly attributed to secondary causes including but not limited to central venous access devices and malignancy [1]. In those who are symptomatic, the clinical presentation ranges from self-limited thrombophlebitis to post-thrombotic syndrome and even life-threatening pulmonary embolism (PE). Treatment modalities include anticoagulation, catheter-directed thrombolysis (CDT), percutaneous mechanical thrombectomy (PMT), or a combination approach, along with management of the underlying etiology. We describe a case of a female with breast cancer and a central venous catheter (CVC) in the left arm who presented with left UEDVT and CVC malfunction and was successfully treated with mechanical thrombectomy to maintain continuous intravenous access.

## Case presentation

A 49-year-old female suffering from right-sided triple-negative breast cancer and on active chemotherapy presented with left arm swelling for the past 10 days. She stated that her left arm swelling had progressed from the elbow up to the shoulder and also complained of heaviness and cramping pain in the left arm (5/10 in severity). Physical examination was remarkable for 4+ edema and tenderness along with a plethora at the level of the shoulder and upper left chest. She was not able to lift her hand above the shoulder and had a restricted range of motion of the left elbow due to swelling. She also had a left-sided CVC which, on assessment, was unable to be flushed or aspirated.

The patient was on an anthracycline-based regimen for her breast cancer for the past year. She had no personal or family history of previous DVT or hypercoagulable state and did not report the use of any hormonal therapy. Differential diagnoses at the time included catheter-associated deep and superficial vein thrombosis or malignant lymphedema.

**Figure 1:**
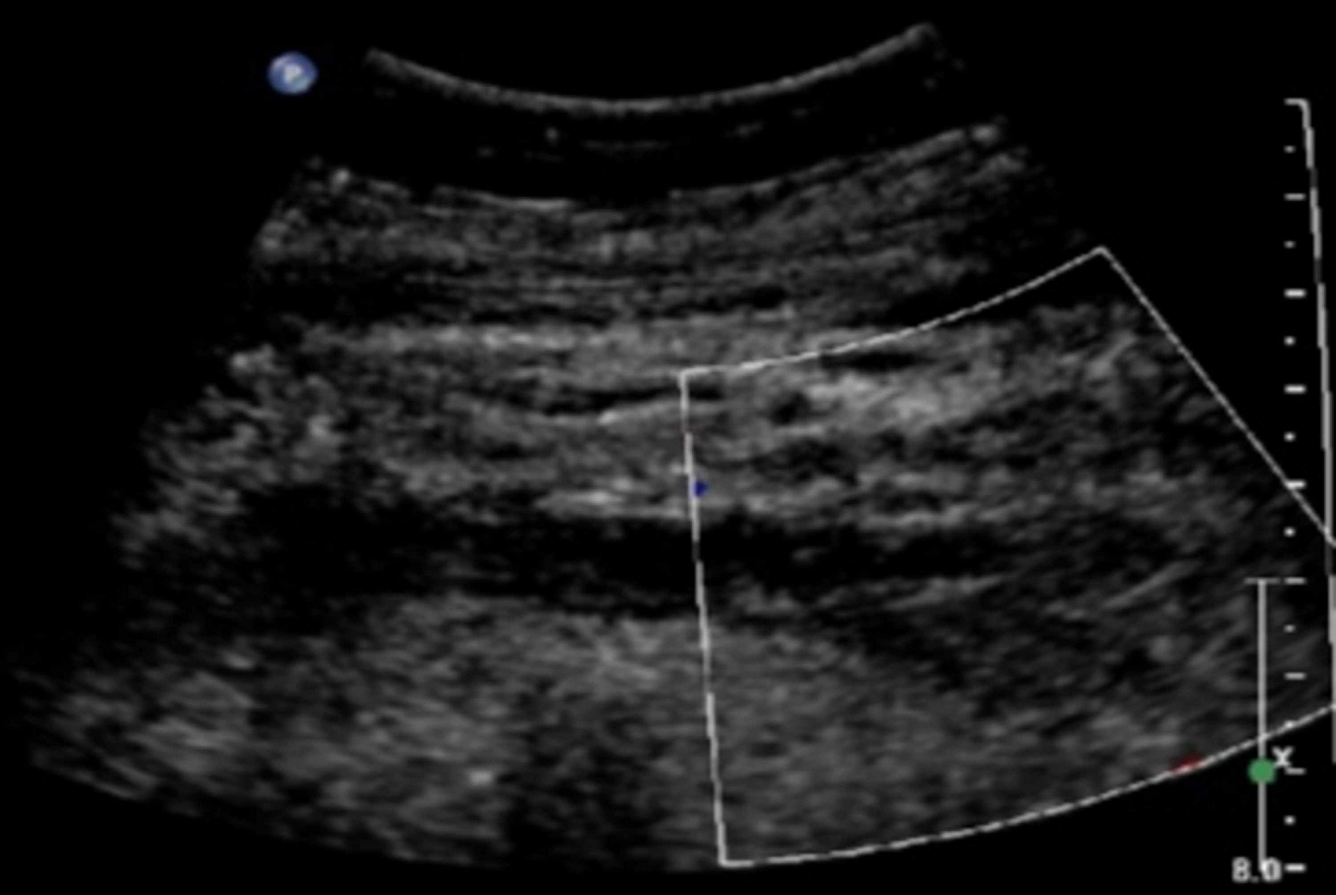
Pre-procedure venous doppler ultrasonography showing the absence of flow in the subclavian vein.

The patient was admitted and preliminary investigations were remarkable for anemia (7.8 g/dl) and leukopenia (3200/mm3). Due to the likelihood of catheter-associated UEDVT, venous duplex ultrasonography was performed which showed acute occlusive DVT of the left subclavian and axillary veins, superficial venous thrombosis of the proximal left basilic, and antecubital veins, and short segment occlusion of the proximal left cephalic vein (Figure 1).

As the diagnosis of catheter-associated UEDVT was confirmed, the patient was started on subcutaneous enoxaparin injection (1 mg/kg) every 12 hours, and the interventional cardiology service was consulted to discuss the need for possible intervention. Considering the patient’s severely progressive symptoms, high thrombotic burden extending from the subclavian to the axillary vein, acute presentation, severe anemia, and the need to treat the CVC malfunction, the decision was made to pursue mechanical thrombectomy with the ClotTriever System (Inari Medical, Irvine, CA).

**Figure 2:**
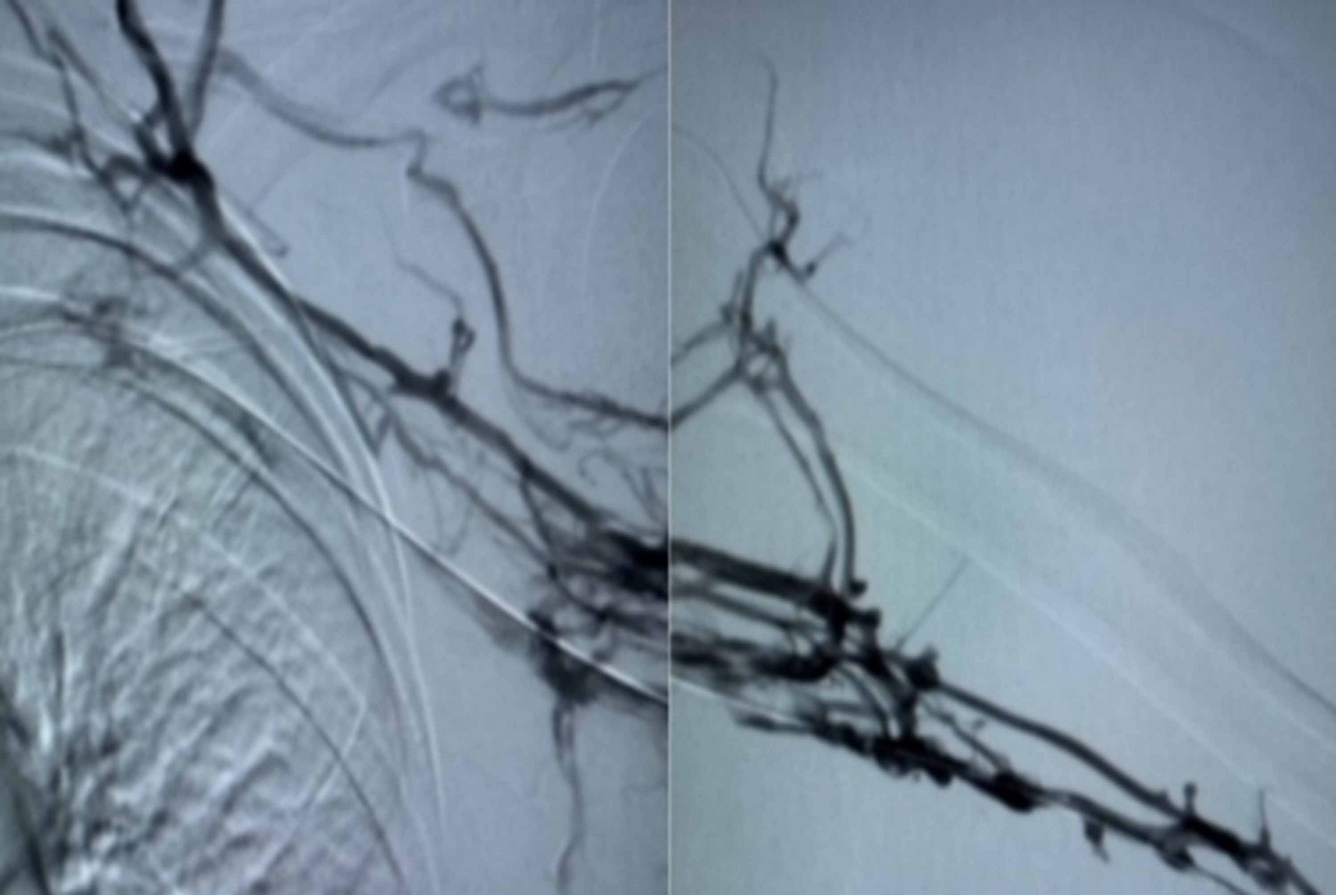
Pre-procedure venography showing thrombosis of the subclavian, axillary, and brachial veins and presence of collaterals.

Left brachial 5Fr venous access was obtained and diagnostic venography was performed. Thrombotic occlusion of the brachial, axillary, and subclavian veins was seen along with collateralization towards the superior vena cava (SVC) and the presence of a large thrombus at the location of the CVC extending into the subclavian vein (Figure 2).

**Figure 3:**
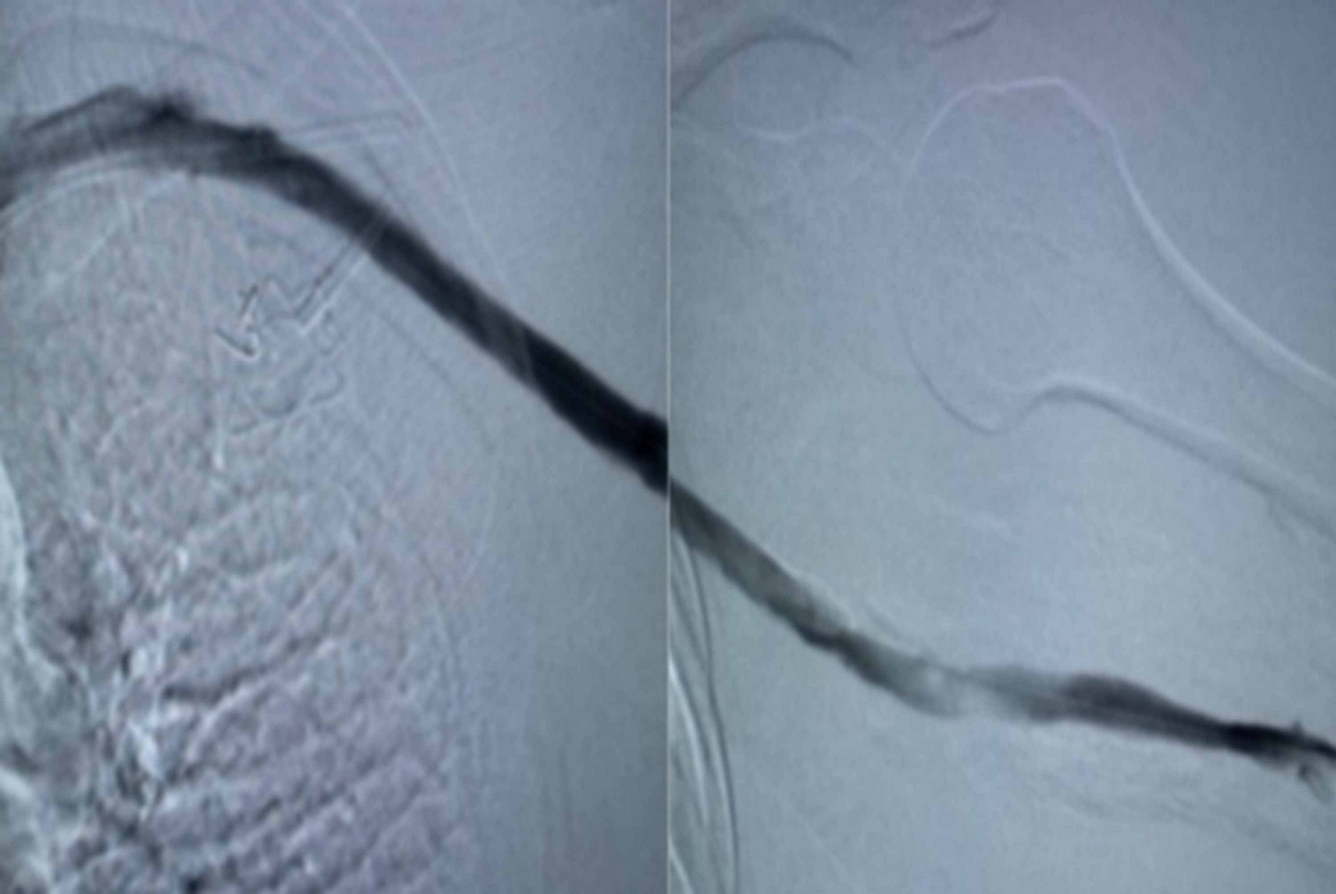
Post-procedure venography showing patent subclavian, axillary, and brachial veins along with the resolution of collaterals.

The occlusion was crossed, with the wire placed in the SVC and further navigated into the inferior vena cava past the right atrium. The wire was exchanged with an Amplatz Super Stiff wire that was placed in the right femoral vein and the ClotTriever 13Fr sheath was inserted into the left brachial vein after pre-insertion venoplasty with a 6.0 mm balloon. The ClotTriever catheter was then inserted through the sheath, advanced beyond the SVC, and deployed. After two passes with the ClotTriever catheter, repeat venography was performed which demonstrated continuous flow from the brachial vein to the subclavian vein. Mild contrast extravasation was noted in the proximal axillary vein and was treated with prolonged low-pressure balloon inflation with a 12 x 40 mm Conquest balloon. Repeat venography demonstrated > 90% patency of the brachial, axillary, and subclavian veins with minimal clot burden, no significant collaterals, and straight-line flow to the SVC (Figure 3).

The patient was monitored overnight in the cardiac telemetry unit and was discharged the next day on full dose subcutaneous enoxaparin with scheduled follow-up after one week. At the follow-up visit, the patient had significant improvement in symptoms along with the resolution of venous engorgement without any sign of bleeding.

## Discussion

The incidence of UEDVT due to CVC is reported to occur in six out of 10,000 hospitalizations, while patients with malignancy have a higher prevalence of 3.8% at 12 months [2]. Serious sequelae of UEDVT are similar to that of lower extremity DVT, including post-thrombotic syndrome and life-threatening PE [3]. Although the incidence of PE is considered to be lower with UEDVT than with lower extremity DVT (LEDVT), a recent study found the incidence of PE to be as high as 15% in ICU patients with UEDVT compared to 8% associated with LEDVT [4]. 

The goals of managing catheter-associated UEDVT include alleviation of symptoms, prevention of embolization, and maintenance of continuous intravenous access. In the absence of contraindications, systemic anticoagulation is recommended for LEDVT, with low-molecular-weight heparin being the preferred choice for patients with malignancy [3]. The American College of Chest Physicians guidelines also recommends anticoagulation over invasive management for UEDVT, with thrombolysis considered in patients with severe progressive symptoms, thrombus extending from subclavian to axillary vein, symptoms < 14 days, life expectancy > one year, and low risk of bleeding [5]. Thrombus removal can also be achieved in DVT through CDT, PMT, or pharmaco-mechanical CDT. However, a major drawback of thrombolysis is its association with clinically significant bleeding [6]. Additionally, the use of CDT requires an extended hospital stay in the ICU as well as serial venography which confers extra hospital costs as well as increased contrast burden on the kidneys, which again should be an important consideration in patients already on chemotherapy. We felt our patient was a good candidate for PMT using ClotTriever due to the potential to clear thrombus without needing thrombolysis.

For patients with indwelling catheters, it is imperative to maintain continuous intravenous access, especially in those with malignancy who require long-term chemotherapy; these patients have a higher incidence of catheter occlusion and resulting malfunction due to the formation of a “fibrin sheath” which might require removal and reinsertion of the catheter, which has its own technical difficulties and higher chances of re-thrombosis [7]. Although the use of fibrinolytic agents has been used to dissolve the fibrin sheath, it does not clear the vein of thrombus and thus it represents a temporary solution to allow for the use of the CVC but does not improve venous patency and associated patient symptoms.

In our case, the ClotTriever System provided a safe, effective, non-thrombolytic option that allowed salvaging the CVC and prevented both serious bleeding complications and the need for extraction and reimplantation of the CVC.

## Conclusions

UEDVT remains an underdiagnosed clinical entity lacking clear guidance for treatment, with no multicenter randomized clinical trials or guidelines addressing the treatment of this condition. Although anticoagulation remains the cornerstone of therapy, interventional therapies without the use of thrombolytic agents appear to be safe and effective as a first-line treatment, especially in patients who require rapid treatment without risking the loss of long-term intravenous access. Our successful outcome using the ClotTriever System in a patient who presented with UEDVT and CVC malfunction further advocates for the use of this non-thrombolytic treatment modality in these settings as a single session interventional procedure.
